# Efficiency of Manchega Sheep Milk Intended for Cheesemaking and Determination of Factors Causing Inefficiency

**DOI:** 10.3390/ani13020255

**Published:** 2023-01-11

**Authors:** Ana Garzón, José M. Perea, Ramón Arias, Elena Angón, Javier Caballero-Villalobos

**Affiliations:** 1 Departamento de Producción Animal, Universidad de Córdoba, 14071 Córdoba, Spain; 2 Centro Regional de Selección y Reproducción Animal de Castilla-La Mancha, Valdepeñas, 13300 Ciudad Real, Spain

**Keywords:** sheep milk, coagulation efficiency, curd yield, deterministic parametric efficiency models, Manchega

## Abstract

**Simple Summary:**

As payment systems currently do not reflect the quality of raw milk intended for cheesemaking, the industry demands tools to estimate an economical value of milk based on its performance during the coagulation process. This study develops algorithms that aim to improve the overall efficiency of the cheese sector through a more adequate connection between the demands of the industry and the technological profiles of raw milk, as well as to establish an objective method to set prices, which particularly consider the usefulness of milk to make cheese. Taking into account that the few studies available on this topic only focus on fresh cheese yield, we also evaluated the moisture of the curd, which led to an algorithm based on dry extract, which is more suitable considering the wide range of cheeses available in the market, and thus, better adjusts to the reality of the industry.

**Abstract:**

Understanding the factors that determine and regulate cheese yield would allow, through deterministic parametric efficiency models, the determination of the most appropriate milk characteristics for the industry, and the estimation of a technological value for these characteristics. The present study aims to evaluate coagulation performance of Manchega sheep milk intended for cheesemaking and explores two models to determine milk technological efficiency. For this purpose, 1200 Manchega sheep milk samples were collected, and analyses were performed for composition, milk coagulation properties (MCP), somatic cell count (SCC), and milk color values. A first model was built based on curd yield (CE) and a second one based on dry curd yield (DCE). GLM and MANCOVA analyses were used to identify the factors that determine curd yield efficiency, which mainly depended on pH, casein, and lactose content and, to a lesser extent, on the speed of coagulation and curd firmness. When comparing both models, differences were linked to the water retention capacity of the curd. Based on this, the DCE model was considered much more accurate for prediction of coagulation efficiency in a wider variety of cheeses, as it does not seem to be affected by moisture loss.

## 1. Introduction

A main aspect of the quality of sheep milk is its ability to be processed into high quality cheese (what is known as ‘milk processing capacity’ or ‘curd yield’), and to obtain a high cheese yield per liter of milk. According to the literature [1], the average value of curd yield for sheep milk is 25.8% (3.9 of milk to obtain 1 kg of cheese) and would not only depend on fat and protein, but also on lactose content. In the Manchega breed, this percentage is actually somewhat lower, standing at around 20% [2]. The quantity and quality of cheese obtained per volume unit of processed milk is directly linked to the benefits of the dairy industry [3]. Therefore, it is essential to know what factors determine the technological efficiency of milk and how they act, in order to obtain a greater economic benefit.

Milk coagulation properties (MCP) have traditionally been considered good indicators for curd yield. However, although there is growing interest in the use of MCP as an indirect measure of curd yield or as a target for genetic improvement of the technological aptitude of milk, the relationship between these parameters is still controversial. For instance, some authors [4] reported that MCP were correlated, although weakly, with moisture-adjusted curd yield, while others [5,6] did not find significant differences between cheeses elaborated from milk with good or bad coagulation performance.

A recent study performed in goat and sheep milk stated that curd yield measures the efficiency of the cheesemaking process and can be predicted based on fat and protein content, variables highly correlated with cheese yield [7]. However, performance and final quality of cheese cannot be attributed solely to composition. According to this author, in the dairy industry, the capacity of milk to produce cheese is generally predicted using mathematical formulas based on fat and protein content. These predictive formulas have been well studied for bovine milk and are very effective, but they may not be the most appropriate for sheep milk, since this kind of milk is characterized by high fat and protein contents, shorter coagulation times, and a low percentage of non-coagulating samples. Furthermore, in sheep, curd firmness has been reported to be practically independent of coagulation time [7]. In-depth knowledge of the factors that determine cheese yield would allow, through efficiency models, determination of the most appropriate characteristics for the industry, and their inclusion in pricing systems in order to guarantee fair prices to producers. The estimation of a value of milk technological traits would enable industry, producers, and regulatory authorities to develop their own production guidelines to guarantee competitiveness and promote sectoral policies that contribute to the global efficiency of a sector of great economic and social interest in Spain.

Caballero-Villalobos et al. [8] assessed the technical efficiency of the coagulation process using a parametric frontier-based approach [9]. This methodological framework was deemed useful to evaluate and optimize the relationship between curd firmness and coagulation time in the manufacture of Manchego cheese. The protein-to-fat ratio and curd yield could lead to two efficiency indicators of the Manchego cheese manufacturing process and, consequently, could be optimized through the technical efficiency methodological framework [10,11]. With this procedure, the most efficient cheese making in a set of experimental units can be used as a primary reference to measure the relative efficiency of each unit and determine the causes of inefficiency. Therefore, this study aims to evaluate coagulation performance of Manchega sheep milk intended for cheesemaking, to determine the factors affecting the coagulation process and to identify those accounting for the inefficiencies. 

## 2. Materials and Methods

### 2.1. Dataset and Sample Collection

The present study included 1200 Manchega sheep milk samples from 4 different flocks affiliated to the Protected Designation of Origin (PDO) Queso Manchego. All samples were obtained from the morning milking in spring and autumn of 2017 and at three time points (early, mid, and late lactation). Only data from 967 (80.58% of the total number) were included in the statistical analysis, as the rest corresponded to milk samples that did not coagulate under standard laboratory conditions and, therefore, no information on curd yield could be obtained. A full description of sample collection, processing, and assessed husbandry factors can be found in Garzón et al. [12]. 

### 2.2. Laboratory Analysis

All analyses were performed in duplicate in the Dairy Small Ruminant Laboratory (Departamento de Producción Animal, Universidad de Córdoba, Spain), and are described in detail in Figueroa Sánchez et al. [13]. Briefly, milk pH was measured with a Crison Basic20 pH meter (Crison Instruments S.A., Barcelona, Spain), and chemical composition of both milk and whey was determined on a MilkoScan FT120 (Foss Electric, Hillerød, Denmark). Coagulation was monitored on a Formagraph viscometer (Foss Electric, Hillerød, Denmark) after adding to 10 mL of milk 50 μL of a 4% dilution of single strength liquid animal rennet dilution (185 IMCU/mL), and values were obtained for rennet clotting time (RCT), curd firming time (k_20_), curd firmness at 60 min (A_60_), and maximum curd firmness (A_max_). Curd yield (CY) was calculated after draining the whey and expressed in milligrams/100 mL of milk and dry curd yield (DCY) was obtained after desiccating the curds in a drying oven at 100 °C for 24 h. Somatic cell count (SCC) was determined on a Fossomatic FC (Foss Electric, Hillerød, Denmark) and a log_10_ transformation was used to express it in somatic cell score (SCS). Finally, color indices of raw milk were measured with a PCE-CSM2 color meter (PCE Instruments, Southampton, UK) using the CIELAB color space, and variables included lightness (L*), red/green value (a*), blue/yellow value (b*), chroma (C*), and hue (h*). 

### 2.3. Modeling Curd Yield Performance of Manchega Sheep Milk

The Cobb–Douglas parametric function was used to model the relationship between yield and fat and protein content in milk [10]. Two models were built: the first one modeled the relationship between CY and fat and protein content, while the second one modeled the relationship of these same independent variables with DCY, according to the following expression:

$$Y_{i}=e^{\alpha }X_{1}^{\beta_{1}}X_{2}^{\beta_{2}}e^{-\mu_{i}}$$

where $Y_{i}$ is the yield (CY or DCY), $X_{1}$ and $X_{2}$ are the independent variables Fat and CP, α is the intercept, $\beta_{1}$ and $\beta_{2}$ are the parameters of the independent variables $X_{1}$ and $X_{2}$, respectively, and $\mu_{i}$ is the term that gathers the non-negative residues ($\mu_{i}\geq 0$).

The adjustment of each model was determined by the adjusted coefficient of determination and the mean absolute error was contrasted using ANOVA. Heteroskedasticity was evaluated using White’s test, and Chow’s test was used to determine the stability of the regression coefficients. The Kolmogorov–Smirnov test was used to verify normal distribution of the residuals, and the Durbin–Watson test was used to detect the absence of autocorrelation of the residuals [8,10]. A description of the models can be found in Table 1.

### 2.4. Curd Yield Efficiency of Manchega Sheep Milk

Once both models were built, and following Greene [14], the deterministic frontier function of each model was calculated using the corrected ordinary least squares method. Previously, the Cobb–Douglas function was linearized by means of a logarithmic transformation, according to the following expression [11]:

$$Y_{i}=e^{\alpha }X_{1}^{\beta_{1}}X_{2}^{\beta_{2}}e^{-\mu_{i}}\to \ln Y_{i}=\alpha +\beta_{1}\ln X_{1}+\beta_{2}\ln X_{2}-\mu_{i}$$

The linearized function is shifted by adding the maximum positive residue to the intercept, thus obtaining the CY and the DCY frontier functions [8].

The frontier functions establish the maximum level of curd or dry curd that can be obtained from 10 mL of milk containing a given combination of fat and protein. The relationship between the observed and estimated yield in each milk sample allows the calculation of an efficiency index according to its yield in curd, or its yield in dry extract of the curd. Therefore, the curd efficiency index (CE), or dry curd efficiency index (DCE), was calculated using the method of Timmer [9], expressed as follows:

$$E_{i}=1-\left(\frac{Y_{i}-Y_{i}^{\prime }}{Y_{i}^{\prime }}\right)$$

where $E_{i}$ is the curd efficiency index (CE), or dry curd efficiency (DCE), $Y_{i}$ is the observed yield (referred to CY or DCY), and $Y_{i}^{\prime }$ is the yield estimated by the frontier function (referred to CY or DCY, respectively).

### 2.5. Determinants of Curd Yield Efficiency of Manchega Sheep Milk

First, the bivariate associations between CE and DCE, and the factors that hypothetically affect the efficiency of the coagulation process were explored. Qualitative factors were analyzed by ANOVA with the Student–Newman–Keuls (SNK) test as a means contrast test, or Student’s *t*-test when the factor only had two levels. Covariate factors were analyzed using Pearson correlations. The variables hypothetically related to the efficiency of the coagulation process are described in Table 2. Efficiency indices (CE and DCE) are fractional data generated by an uncensored process; therefore, models based on ordinary least squares regression are appropriate to analyze the determinants of inefficiency [8,15,16]. Causes of inefficiency were studied from both a univariate and a multivariate approach, simultaneously considering CE and DCE.

Multivariate analysis of variance with factors and covariates (MANCOVA) was used to identify the factors that determine curd yield efficiency, simultaneously considering the two dependent variables (CE and DCE) when performing the statistical test [17]. MANCOVA offers a general test of the equality of the mean vectors for several groups, but it cannot establish significant differences between groups from their mean vectors [18]. The MANCOVA model was built following a backward stepwise sequence. Wilks’ λ test, Pillai’s trace, and Lawley–Hotelling trace were used as contrast statistics [19]. The initial model included the main effects of all fixed, random, and covariate factors listed in Table 2. At each step, the variable with the highest critical value is removed from the model. This sequence is repeated until all the model variables have a critical value of less than 5% in at least one test statistic. Previously, normality, linearity, and homogeneity of the variance covariance matrices, and multicollinearity were evaluated [20].

The general linear model (GLM) was used to analyze the factors that determine curd yield efficiency considering each dependent variable (CE and DCE) independently. This model is based on an ordinary least squares regression approach to describe the relationship between one or more predictors (which can be factors or covariates) and a continuous response variable [21]. The model is expressed as follows:

$$E=\overset{n}{\underset{i=1}{\sum }}\beta_{i}f_{i}+\delta$$

where E is the curd yield efficiency measure (CE or DCE), $\beta_{i}$ are the unknown parameters to be estimated, $f_{i}$ are the fixed, random or covariate factors, and δ corresponds to the error term. GLM models were built following a manual step-by-step sequence, considering the main effects of all fixed, random, and covariate factors listed in Table 2. 

A value of *p* < 0.05 was used as the retention criterion. As a first step, all possible single-variable models were compared using the Akaike Information Criterion (AIC) value. All remaining factors are added one by one to the model with the lowest AIC value and a predictor and compared based on the AIC value. This process was repeated until the model with the lowest AIC was obtained [22]. This was considered the most plausible and was selected as the final model. Once the model was defined, the degree of collinearity was analyzed using the variance inflation factor (VIF) of the regression coefficients. A serious multicollinearity problem was considered to exist if any of the VIFs were greater than 10 [23]. The Kolmogorov–Smirnov test was used to verify normal distribution of the residuals, Durbin–Watson test to detect the absence of autocorrelation of the residuals, and heteroscedasticity was evaluated using White’s test. The fit of the models was determined using the adjusted coefficient of determination, the mean absolute error, and the mean absolute percentage error [10,11].

All data were analyzed using the statistical software Statgraphics Centurion XV (StatPoint Technologies Inc., Warrenton, VA, USA).

## 3. Results and Discussion

### 3.1. Efficiency of the Coagulation Process

After using the Cobb–Douglas function, CY and DCY showed a good fit to fat and protein, although DCY fits much better than CY (Table 1). As expected, adjusting to the dry matter content of the curd reduces the variability of the output and, consequently, improves the fit of the function. Returns to scale measures the variations in production due to a proportional change in the factors, which in the Cobb–Douglas function are given by the elasticities β_1_ and β_2_ for fat and protein content [24]. As the sum of elasticities is less than 1, the production of curd is carried out with decreasing returns to scale; that is, if fat and protein content each increase by 1%, an extra amount of curd of less than 1% would be obtained. However, the sum of elasticities is practically 1 in the DCY model, indicating that the production process measured in dry matter follows constant returns to scale; that is, the percentage variations in both factors generate the same percentage increase in dried curd. These findings are relevant in order to economically optimize the cheesemaking process. If the important thing is to obtain solids, the combination of fat and protein that will minimize the cost of production will depend only on the price ratio of fat and protein, being independent of the level of production. If, by contrast, the product is measured fresh, then the volume of production should also be considered [24].

**Figure 1:**
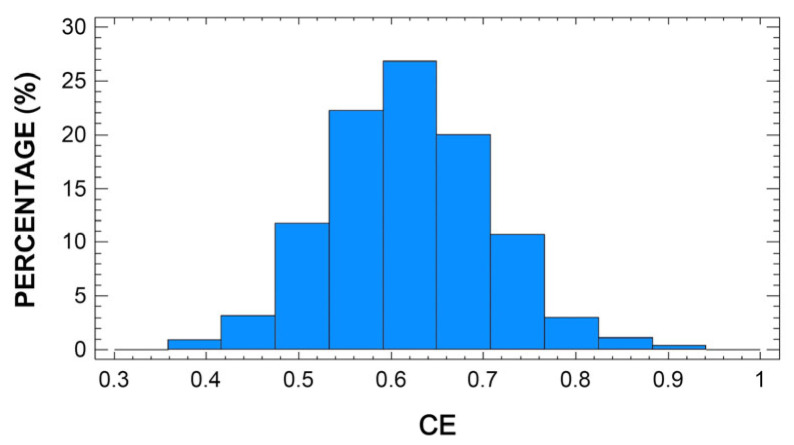
Frequency distribution (histogram) of curd efficiency (CE) in Manchega sheep milk (*n* = 967).

**Figure 2:**
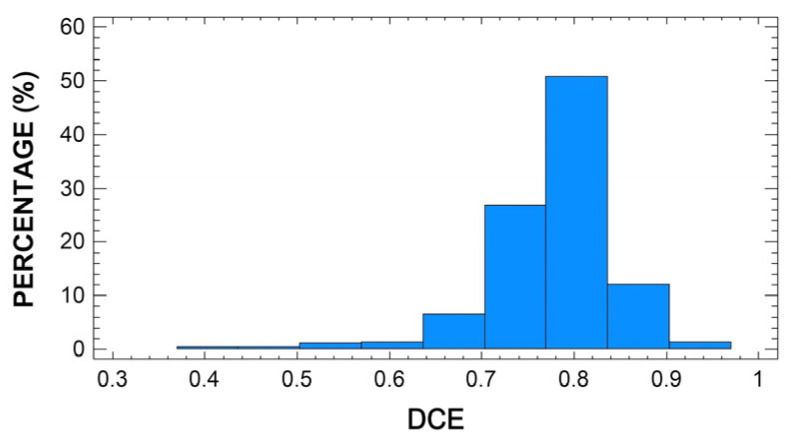
Frequency distribution (histogram) of dry curd efficiency (DCE) in Manchega sheep milk (*n* = 967).

Figure 1 and Figure 2 show the distribution of frequencies obtained for CE and DCE, respectively. The Kolmogorov–Smirnov test proved that both variables adjust to a Gaussian distribution (*p* > 0.05). The average CE was 0.62, with a standard deviation of 0.09. The minimum and maximum efficiency were 0.39 and 1.00, respectively. About 25% of the sheep included in this study were producing milk at a curd efficiency level below 0.56, while another 25% exceeded an efficiency level of 0.67. By comparison, the average DCE was 0.78, with a standard deviation of 0.06. The minimum and maximum efficiency were 0.40 and 1.00, respectively. Most of the studied sheep were producing milk with efficiency levels within the range 0.73 to 0.88. About 3% of the sheep produced milk with dry curd efficiency levels of less than 0.50%. There are very few published studies focusing on modeling the efficiency of sheep milk performance based on other related parameters. Thereby, it is worth highlighting the paper by Caballero-Villalobos et al. (2018), which established an efficiency model for curd firmness based on coagulation time. The average value for efficiency estimated in the forementioned work is 0.69, with a minimum of 0.33 and a maximum of 1.00. These values are fairly similar to those reported for the present study. Nonetheless, no further bibliography was found to compare or discuss these results. 

Table 3 displays the values of milk traits that maximize curd yield. The maximum CE (49.57 g/100 mL of milk) was obtained with 9.47% fat and 4.89% protein, while the maximum DCE (16.75 g/100 mL of milk) was obtained with 7.81% fat and 6.35% protein. It is worth mentioning that the highest CE was obtained with a 34.65% of moisture in the curd while, for DCE, this value was found to be somewhat lower (10%). It can also be observed that both models differ in other husbandry factors that maximize efficiency, highlighting stage of lactation (SOL), occurrence of syneresis, and season of lactation (Season). Furthermore, the time-related coagulation parameters link higher CE with greater vales of RCT and k_20_, which are indicative of a slower coagulation process.

**Figure 3:**
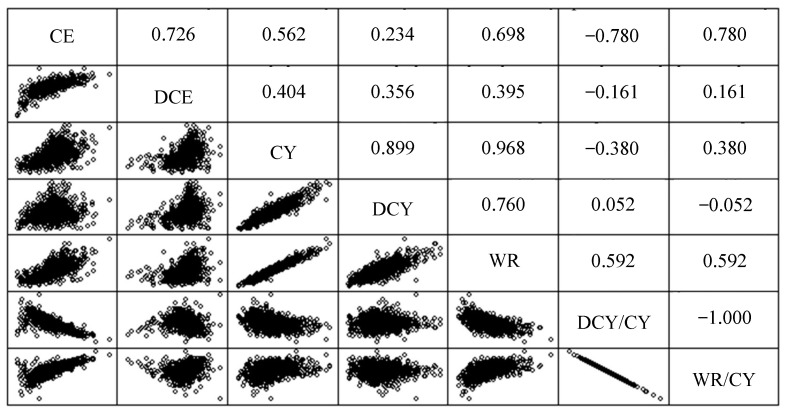
Frequency distribution of curd efficiency (CE) and dry curd efficiency (DCE) in Manchega sheep milk (*n* = 967).

Figure 3 represents the bivariate correlation pattern between variables related to curd yield. The positive correlations between DCY, water retained in the curd (WR), and CY are obvious and evident because the sum of the first two variables results in the latter. However, CY is more strongly correlated with WR than with DCY, and the dispersion pattern contains less variability. Correlations between these variables and the ratio of water (WR/CY) and dry matter (DCY/CY) in curd evidence that WR/CY tends to increase with CY (and DCY/CY tends to decrease proportionally). Therefore, from a technological point of view, this increase in curd yield due to higher water retention should not be considered a “true” improvement, since the higher yield is due to an increment of the percentage moisture and not an increase in cheese solid components. CE and DCE are positively correlated. However, the impact of DCE on CE is more intense at low levels of CE than at high levels, as suggested by the dispersion pattern. This means that the influence of milk composition (DCY) is more important at lower CE and that higher levels of CE are due to a greater retention of water in the curd. The evidence of correlation between CE and DCE has methodological implications for the study of causes of inefficiency. According to Wang et al. [19], if the specific correlation pattern between two or more dependent variables is not taken into account, this test may not be able to identify significant effects. At a practical level this can be solved by complementing the typical unidirectional approach for each dependent variable with a multivariate approach in the analysis of variance that can help differentiate, in a more specific way, which factors optimize coagulation efficiency, as well as the parameters that determine its inefficiency.

### 3.2. Factors Affecting the Coagulation Process

#### 3.2.1. Bivariate Associations

**Figure 4:**
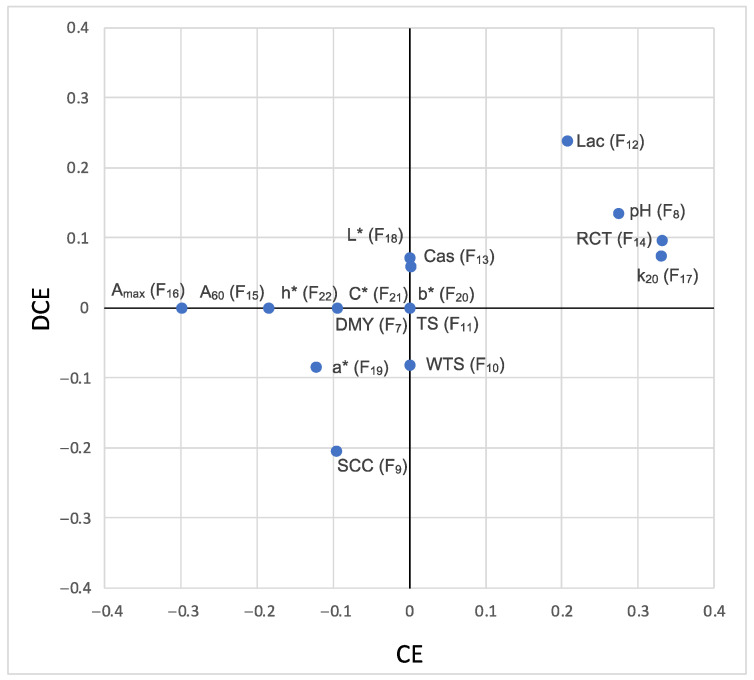
Bivariate associations (Pearson correlations) between curd efficiency (CE) and dry curd efficiency (DCE) and the quantitative factors evaluated (*n* = 967).

**Figure 5:**
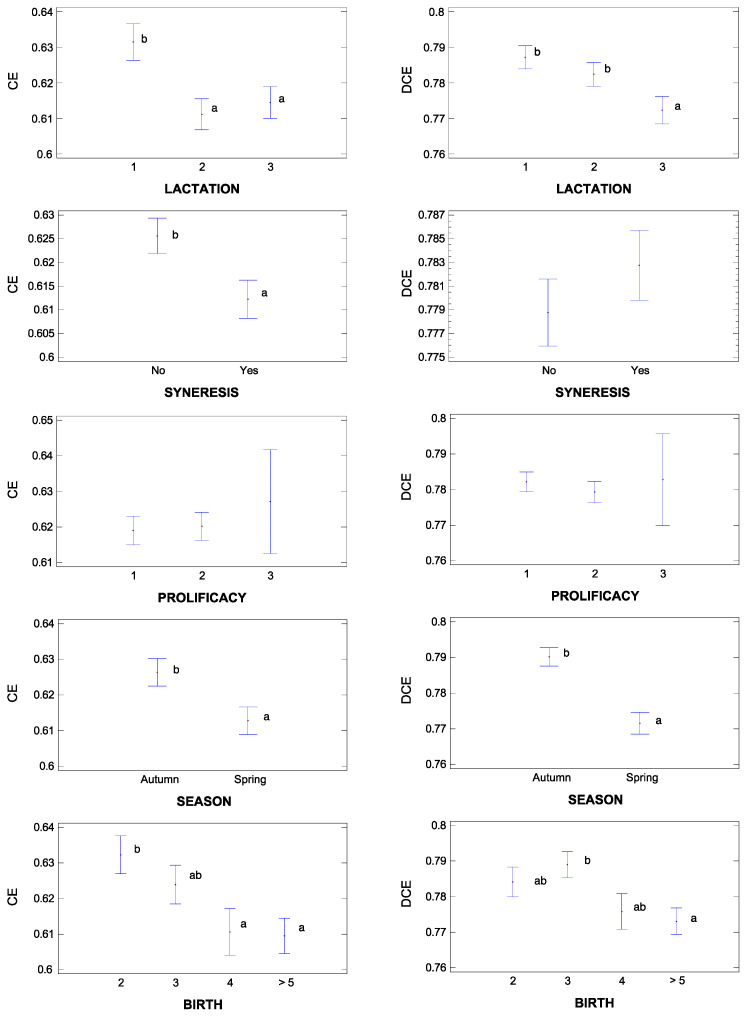
Bivariate association (ANOVA or Student’s *t*) between curd efficiency and the categorical factors evaluated (mean ± standard error). Left column = CE, right column = DCE. “a”, “b”, and “ab”: Means without a common letter are significantly different at *p* < 0.05 according to Student’s *t* test or SNK *post hoc* test (*n* = 967).

The quantitative and categorical variables included in the bivariate analysis are presented in Figure 4 and Figure 5, respectively. Of the 18 covariable factors analyzed, only 5 and 2 were significantly correlated with CE and DCE, respectively, although this association was fairly weak (Figure 4). DCE was negatively correlated with SCS and positively with lactose content, the latter being also positively correlated with CE. The strongest correlations were observed between CE and variables related to coagulation process: positives for pH, k_20_, and RCT, and negative for A_max_.

In both models, a positive correlation of efficiency with lactose content was observed (r = 0.21 for CE and r = 0.24 for DCE), which is in line with the results published for sheep [25] and for goat [26], where it was reported that an increase in lactose levels is linked to an improvement of curd yield. As low lactose contents have been reported to be indicative of poor udder health conditions, the aforementioned studies also support the negative correlation observed between DCE and SCS (r = −0.20), as well as a negative tendency between DCE and WTS (r = −0.08), since an increase in somatic cell levels reduces lactose concentration content, consequently increasing the loss of nutrients during curd draining and reducing yield. 

According to studies performed in dairy cows [3] and studies comparing dairy cows and sheep [1], milk coagulation properties and curd yield are not so much affected by milk composition as expected, which is evident in the DCE model. However, the CE model shows that the most efficient milk samples correspond to those with a more alkaline pH (r = 0.27), longer RCT (r = 0.33), higher k_20_ (r = 0.33), and lower A_max_ (r = −0.30). These values would correspond to milk with “worse” coagulation parameters, resulting in a softer and more hydrated curd and poor drainage. Again, the CE model seems to be conditioned by the percentage of moisture retained in the curd, in agreement with Johnson et al. [27], who associated slow RCTs with an increase in moisture in the curd.

Three categorical factors were significantly associated with CE and DCE (*p* < 0.05): season of lambing (Season), stage of lactation (SOL), and parity number (Parity) (Figure 5). These three factors affect CE and DCE in the same direction.

Thus, autumn lactations show improved CE and DCE compared to spring lactations. By all means, as sheep are seasonal animals [28], shorter days lead to a decrease in milk yield, resulting in higher concentrations of fat and protein and, as a consequence, in an improvement in milk’s performance.

Secondly, a lower efficiency is observed as lactation progresses. This result is contrary to that reported by other authors who described that, as lactation progresses and the amount of milk ordered decreases, there is a concentration effect of milk components [29] and, as a consequence, an improvement in performance [30]. However, our results are in line with other authors [31,32], who measured a progressive decrease in lactose content as lactation progresses, associating it with a worsening of the health status of the udder and, as a consequence, with a reduced efficiency. This same interpretation would apply to parity. According to Vacca et al. [25], SCS increases with parity, due to a deterioration of the udder related to decreasing contents of lactose in milk. 

The occurrence of syneresis significantly affected CE (*p* < 0.05) but not DCE, diminishing efficiency. This may be due to the fact that the syneresis process is nothing but a rapid draining of the curd [33] and, as mentioned before, the CE model is determined to a large extent by the retention of water in the curd. Overall, differences between both efficiency indicators could be based on the phenomenon of water retention in CE, which is obviously separated from DCE. In any case, it would be very difficult to obtain clear conclusions with this type of analysis, fundamentally due to the existing interactions between the different variables studied. For this reason, other statistical analyses have been carried out to give greater clarity to the results.

#### 3.2.2. Multivariate Analysis of Covariance (MANCOVA)

MANCOVA analysis was used to identify the factors that determine yield efficiency, considering, simultaneously, the two variables (CE and DCE) and their pattern of correlation [20]. As the test statistics used yielded monotonous results, only those obtained with Wilks’ λ test are shown. The best results were obtained with the model shown in Table 4. 

The variables TS, RCT, A_60_, and C* were not included in this model, as they strongly intensify multicollinearity. Eight variables were found to be significant factors for both CE and DCE coagulation efficiency models. The λ and F statistics suggest that the main factor causing inefficiency is Lac, followed by k_20_ and A_max_. Cas, initial pH, and Season were also identified as causes of inefficiency, although they have a lesser impact. Lastly, and with a moderate effect, the model highlights Flock and SCS. The set of variables that most impacts the efficiency of the coagulation process is chemical composition (Lac and Cas), followed by coagulation properties (A_max_ and k_20_), and finally the hygienic-sanitary quality of the milk (pH and SCS). Season had a moderate effect, while none of the colorimetric variables were significant.

#### 3.2.3. Generalized Linear Models (GLM)

Causes of inefficiency were analyzed using GLM, considering each dependent variable (CE and DCE) separately. Table 5 shows the results obtained for CE. The adjusted coefficient of determination reached a value of 22.5% and the mean absolute error was 0.06, so the model fit is considered poor, probably due to the great variability in coagulation efficiency because of the interactions between the studied parameters. The VIF of the regression coefficients fluctuated between 1.2 and 2.4, so there is no multicollinearity problem. The Kolmogorov–Smirnov test proved that the residuals adjust to a Gaussian distribution (*p* > 0.05). In addition, White’s test did not evidence heteroscedasticity (*p* > 0.05) and the Durbin–Watson test (*p* > 0.05) indicated no autocorrelation. Thus, the data were considered suitable for GLM analysis. GLM showed that seven out of twenty-four factors significantly affected curd yield efficiency. The *F* statistics highlighted the following variables as the most important determinants of CE: Lac, A_max_, Casein, and initial pH. Other variables such as Season, Parity, and Flock showed a more moderate impact. Regression coefficients revealed that spring lactations, second and third lambing, pH, lactose content, and casein content had a positive effect on CE. Contrastingly, autumn lactations, high parity numbers (≥4), and A_max_ showed a negative effect.

Table 6 reports GLM results obtained for DCE. The adjusted coefficient of determination reached a value of 15.5%, and the mean absolute error was 0.04, so the model fit is considered poor. The VIF of the regression coefficients fluctuated between 1.2 and 1.8, evidencing no multicollinearity problem. The Kolmogorov–Smirnov test showed that the residuals fit a Gaussian distribution (*p* > 0.05). In addition, White’s test did not show heteroscedasticity (*p* > 0.05) and the Durbin–Watson test (*p* > 0.05) indicated no autocorrelation. GLM showed that six factors significantly affected dry curd yield efficiency of Manchega sheep’s milk. The *F* statistics revealed the most important determinants of DCE were Lac, Cas, and pH of milk and, to a lower extent, k_20_ and SCS. Regression coefficients revealed a positive effect of pH, Lac, and Cas on DCE, while SCS and k_20_ seemed to have a negative effect.

Both models indicate that there seems to be an improvement in efficiency when pH increases. However, it is well known that the pH of milk has a great impact on coagulation, since higher values slow the coagulation process. Taking into account that the average pH value in the present study was 6.61, we believe that this improvement in efficiency is not due to a significant alkalinization of milk. Rather, we believe that, unlike other studies that reported greater efficiency in samples with a pH close to 6.5, coagulation is favored by slight increases in pH that bring the values to the normal pH range of milk.

Likewise, other authors observed in goat milk that slightly higher pH values (and similar to our mean values) are related to a greater recovery of nutrients in the curd [26]. In addition, the increase in lactose content (responsible for osmotic regulation in milk) [34] and casein (active component in the coagulation process) [35] boost efficiency in both models. However, the two models are contradictory when it comes to the influence of coagulation parameters. In this way, CE increases when the firmness of the clot is lower, evidencing the effect of water retention in the curd for this model, while DCE increases with higher curd firming times, which result in a faster draining. This is consistent with Cipolat-Gotet et al. [36], who concluded that curd moisture contributes significantly to individual CY variability. Finally, it should be noted that, contrastingly with the CE model, DCE was not affected by any of the categorical variables related to the progress of lactation and a resulting progressive deterioration of the udder, which would be evidenced by a worsening of coagulation parameters, as was observed in the case of CE.

#### 3.2.4. Causes of Inefficiency

Table 7 summarizes the determinants of efficiency obtained with both methodologies. Four variables were identified as common causes of inefficiency for CE and DCE by both the GLM and MANCOVA models: Flock, pH, lactose content, and casein content. These factors also affect CE and DCE in the same way. The efficiency of the curd development process improves with greater lactose and casein contents, and pH of milk within normal ranges. Causes of inefficiency for DCE identified by GLM and MANCOVA included SCS and coagulation rate. However, for CE, only A_max_ was evidenced as a cause of inefficiency.

Of all studied variables, high lactose content is the main determinant of curd yield performance, leading to an increase in both CE and DCE. This is in alignment with results obtained by Vacca et al. [25], who observed that a decrease in the percentage of lactose in milk induces a lower aggregation of protein in the curd, leading to a greater loss of nutrients in the whey and, consequently, to a poorer performance. Regarding categorical variables, it is worth highlighting the effect of Flock on both efficiency indices, although to a lesser extent on CE. As discussed in Garzón et al. [12], this effect is very difficult to define, since, according to other authors [37], it involves many factors (including variables such as management, nutrition, hygiene, milking routine, etc.), which can directly affect the characteristics of the milk and, therefore, its performance and efficiency.

## 4. Conclusions

Curd yield (measured as fresh or dry) fits well to the Cobb–Douglas function, and deterministic parametric efficiency models have proven to be useful to determine the factors that influence coagulation in sheep milk. Outcomes from the present study revealed that the two explored models (CE and DCE) were similarly affected not only by physicochemical traits such as pH, low lactose content, and low casein content, but also by husbandry factors such as flock, which were all identified as the main causes of inefficiency. However, differences in coagulation efficiency were found between both models, and were mainly linked to the amount of moisture captured within the curd. Traditionally, curd yield has been widely used to as an indicator for the performance of milk coagulation, but depending on the variety of cheese, this trait may be distorted, since final cheese yield can be significantly reduced due to moisture loss during ripening. By comparison, efficiency models built using dry curd yield (DCE) adjust better, since they are not affected by the water capacity retention of the curd. Therefore, we consider that DCE model is much more accurate for the prediction of coagulation efficiency in a wider variety of cheeses. Nevertheless, the impact of curd moisture on the technological performance of milk should be studied in greater depth.

## Figures and Tables

**Table 1:** Adjusted regression models between the observed values (Y) for curd yield (CY) and dry curd yield (DCY), and fat ($X_{1}$) and protein ($X_{2}$) content of Manchega sheep milk (*n* = 967).

Model	Parameter	Coefficient	S.E.
Curd yield (CY)			data
	α	5.3885	0.2765
	β_1_	0.4833	0.0185
	β_2_	0.4106	0.0351
	White test	0.831	
	Chow test	0.948	
	Kolmogorov–Smirnov test	0.432	
	Durbin–Watson test	0.064	
	ANOVA	<0.001	
	Adjusted R^2^	61.97	
	Mean absolute error (MAE)	2.93	
Dry curd yield (DCY)			
	α	1.8721	0.0597
	β_1_	0.4804	0.0116
	β_2_	0.5171	0.0221
	White test	0.949	
	Chow test	0.567	
	Kolmogorov–Smirnov test	0.231	
	Durbin–Watson test	0.770	
	ANOVA	<0.001	
	Adjusted R^2^	82.98	
	Mean absolute error (MAE)	0.65	

**Table 2:** Description of the variables used to estimate yield efficiency and causes for inefficiency in Manchega sheep milk (*n* = 967).

Variable	Description	Units	Mean	S.D.
Yield efficiency				
CY (Y_1_)	Curd yield	g/100 mL	26.80	6.10
DCY (Y_2_)	Dried curd yield	g/100 mL	11.18	2.34
WR (Y_3_)	Water retention (CY-DCY)	g/100 mL	15.71	4.11
Fat (X_1_)	Fat content in milk	g/100 mL	6.48	1.84
CP (X_2_)	Protein content in milk	g/100 mL	5.69	0.81
Random factors				
Flock (F_1_)	Flock of origin	1 to 4	-	-
Fixed factors				
SOL (F_2_)	Stage of lactation	1 to 3	-	-
Syneresis (F_3_)	If A_60_ < A_max_	Yes or no	-	-
Prolificacy (F_4_)	Number of lambs	1 to 3	-	-
Season (F_5_)	Season of lambing	Autumn or spring	-	-
Parity (F_6_)	Lactation number	2 to 5 or more	-	-
Covariable factors				
DMY (F_7_)	Daily milk yield	mL	1.093	474.3
pH (F_8_)	pH	−log[H^+^]	6.61	0.29
SCS (F_9_)	Somatic cell score	log_10_ (10^3^ cells/mL)	5.24	0.62
WTS (F_10_)	Whey total solids	%	11.19	2.47
TS (F_11_)	Milk total solids	g/100 mL	17.95	2.36
Lac (F_12_)	Lactose content in milk	g/100 mL	4.88	0.38
Cas (F_13_)	Casein content in milk	g/100 mL	4.51	0.69
RCT (F_14_)	Rennet clotting time	min	21.39	11.48
A_60_ (F_15_)	Curd firmness at 60 min.	mm	38.26	11.11
A_max_ (F_16_)	Maximum curd firmness	mm	42.14	9.16
k_20_ (F_17_)	Rate of curd aggregation	min	24.72	12.40
L* (F_18_)	Lightness	[0, 100]	82.66	2.34
a* (F_19_)	Red/Green value	[−60, +60]	−2.55	0.81
b* (F_20_)	Blue/Yellow value	[−60, +60]	5.51	1.65
C* (F_21_)	Chroma or saturation	(a*^2^ + b*^2^)^1/2^	4.67	2.05
h* (F_22_)	Hue	tan^−1^ (b*/a*)	−0.55	0.28

**Table 3:** Traits that optimize coagulation efficiency in Manchega ewe milk (*n* = 967).

Variable	CE	DCE
CY (Y_1_)	49.57	26.76
DCY (Y_2_)	14.92	16.76
WR (Y_3_)	34.65	10.00
Fat (X_1_)	9.47	7.81
CP (X_2_)	4.89	6.35
Flock (F_1_)	4	1
SOL (F_2_)	1	3
Syneresis (F_3_)	No	Yes
Prolificacy (F_4_)	1	1
Season (F_5_)	Spring	Autumn
Parity (F_6_)	3	3
DMY (F_7_)	1.420	1.300
pH (F_8_)	6.61	6.56
SCS (F_9_)	5.95	6.12
WTS (F_10_)	1.79	1.71
TS (F_11_)	15.36	20.02
Lac (F_12_)	4.88	4.94
Cas (F_13_)	3.93	5.12
RCT (F_14_)	19.45	5.30
A_60_ (F_15_)	50.0	47.76
A_max_ (F_16_)	50.0	54.26
k_20_ (F_17_)	22.15	7.00
L* (F_18_)	83.19	85.00
a* (F_19_)	−2.37	−2.32
b* (F_20_)	4.25	0.80
C* (F_21_)	4.86	2.45
h* (F_22_)	4.22	1.90

**Table 4:** Determinants for curd efficiency (CE and DCE), according to MANCOVA (*n* = 967).

Variable	Wilks λ	*F*	*p*
Flock (F_1_)	0.949	8.49	<0.001
SOL (F_2_)	-	-	>0.05
Syneresis (F_3_)	-	-	>0.05
Prolificacy (F_4_)	-	-	>0.05
Season (F_5_)	0.973	13.22	<0.001
Parity (F_6_)	-	-	>0.05
DMY (F_7_)	-	-	>0.05
pH (F_8_)	0.977	11.62	<0.001
SCS (F_9_)	0.981	9.22	<0.001
WTS (F_10_)	-	-	>0.05
TS (F_11_)	-	-	>0.05
Lac (F_12_)	0.935	33.71	<0.001
Cas (F_13_)	0.960	20.18	<0.001
RCT (F_14_)	-	-	>0.05
A_60_ (F_15_)	-	-	>0.05
A_max_ (F_16_)	0.964	18.12	<0.001
k_20_ (F_17_)	0.941	30.52	<0.001
L* (F_18_)	-	-	>0.05
a* (F_19_)	-	-	>0.05
b* (F_20_)	-	-	>0.05
C* (F_21_)	-	-	>0.05
h* (F_22_)	-	-	>0.05

**Table 5:** Determinants for curd efficiency (CE) according to GLM (*n* = 967).

Factors	Coefficients	S.E.	F Value	*p* Value	FIV
Random factors					
Flock (F_1_)	-	-	8.99	<0.001	1.7
Fixed factors					
Season (F_5_)	-	-	14.87	<0.001	1.7
Spring	0.012	0.0032	-	-	-
Autumn	−0.012	0.0032	-	-	-
Parity (F_6_)	-	-	2.62	0.049	1.7
2	0.0074	0.0042	-	-	-
3	0.0068	0.0043	-	-	-
4	−0.0069	0.0049	-	-	-
≥ 5	−0.0073	0.0044	-	-	-
Covariable factors					
pH (F_8_)	0.0952	0.0174	29.85	<0.001	1.3
Lac (F_12_)	0.0763	0.0099	58.96	<0.001	2.4
Cas (F_13_)	0.0341	0.0052	42.88	<0.001	2.1
A_max_ (F_16_)	−0.0017	0.0002	49.38	<0.001	1.2

**Table 6:** Determinants for dry curd efficiency (DCE) according to GLM (*n* = 967).

Factors	Coefficients	S.E.	F Value	*p* Value	FIV
Random factors					
Flock (F_1_)	-	-	9.00	<0.001	1.7
Covariable factors					
pH (F_8_)	0.0756	0.0161	21.99	<0.001	1.8
SCS (F_9_)	−0.0045	0.0014	9.56	0.002	1.2
Lac (F_12_)	0.0541	0.0065	68.32	<0.001	1.7
Cas (F_13_)	0.0185	0.0033	31.27	<0.001	1.5
k_20_ (F_17_)	−0.0007	0.0002	18.23	<0.001	1.8

**Table 7:** Summary of the determinants for curd efficiency (CE and DCE) according to MANCOVA and GLM (*n* = 967).

	MANCOVA	CE (GLM)		DCE (GLM)	
Variable	F	F	Effect	F	Effect
Flock (F_1_)	8.49	5.58	-	13.30	-
Season (F_5_)	13.22	8.48	Spring > Autumn	ns	-
Parity (F_6_)	ns	2.93	2 and 3 > 4 and 5 or more	ns	-
pH (F_8_)	11.62	25.92	Positive	21.99	Positive
SCS (F_9_)	9.22	ns	-	9.56	Negative
Lac (F_12_)	33.71	68.18	Positive	68.32	Positive
Cas (F_13_)	20.18	30.01	Positive	31.27	Positive
A_max_ (F_16_)	18.12	51.10	Negative	ns	-
k_20_ (F_17_)	30.52	ns	-	18.23	Negative

ns = non-significant.

## Data Availability

The data presented in this study are available upon request from the corresponding author.
